# SCREENING FOR LOW SHORT PHYSICAL PERFORMANCE BATTERY SCORES: CAN GRIP STRENGTH AND SINGLE CHAIR STAND BE USEFUL?

**DOI:** 10.1093/geroni/igz038.2510

**Published:** 2019-11-08

**Authors:** Paulo H Chaves

**Affiliations:** Benjamin Leon Center for Geriatric Research and Education at Florida International University, Miami, Florida, United States

## Abstract

The clinical value of low Short Physical Performance Battery (SPPB) scores for identification of older adults at-disability-risk who may benefit from structured intervention is well-established. Feasibility concerns – e.g., time, space constraints – are factors that often preclude SPPB implementation in clinical settings. We assessed whether grip strength (GS) and/or single chair stand (SCS), simple and highly feasible tests, could be useful for clinical identification of older adults with poor SPPB performance. Cross-sectional study using most recent data (Round 7) from the National Health and Aging Trends Study, which enrolled a large U.S. representative sample of Medicare beneficiaries 65 years and older (baseline round: 2011; yearly follow-ups). Nursing home residents were excluded. Sample size was 4,612. Outcome: poor SPPB performance (score <8). Low GS: <20 Kg (women) or <30 Kg (men), and able to do a SCS without use of arm (yes/no) were predictors. Logistic regression, areas under the curves (AUC), and accuracy statistics were computed. AUC for low GS was 0.66, and SCS inability was 0.68; when both tests were considered together, AUC increased significantly: 0.76. Among those SCS-unable (n=752), 95.6% had SPPB<8. A two-stage screening approach; i.e., detection of SCS inability first, followed by low GS in those SCS-able resulted in a net-sensitivity of 75.3%, and net-specificity of 83.5%. Sequential screening with SCS and GS testing might offer a case finding screening approach appealing to busy clinical settings from feasibility, accuracy, and/or efficiency perspectives for identification of older adults with low SPPB who may benefit from established interventions.

